# Educational gradients in the use of electronic cigarettes and heat-not-burn tobacco products in Japan

**DOI:** 10.1371/journal.pone.0191008

**Published:** 2018-01-12

**Authors:** Yuki Miyazaki, Takahiro Tabuchi

**Affiliations:** 1 School of Medicine, Osaka University, Suita, Osaka, Japan; 2 Center for Cancer Control and Statistics, Osaka Medical Center for Cancer and Cardiovascular Diseases, Higashinari-ku, Osaka, Japan; Legacy, Schroeder Institute for Tobacco Research and Policy Studies, UNITED STATES

## Abstract

**Objectives:**

In addition to electronic cigarettes (e-cigarettes), tobacco companies have recently begun to sell heat-not-burn tobacco products, Ploom and iQOS in Japan. Previous research has reported an inverse association between combustible cigarette smoking and educational attainment, but little is known about the association for e-cigarettes, especially heat-not-burn tobacco products. Our objective was to analyze the relationship between educational attainment and e-cigarette and heat-not-burn tobacco use.

**Setting:**

An internet survey (randomly sampled research agency panelists) in Japan.

**Participants:**

A total of 7338 respondents aged 18–69 years in 2015 (3632 men and 3706women).

**Primary measures:**

Adjusted odds ratios (ORs) of educational attainment for current smoking (combustible cigarettes), e-cigarette ever-use, and heat-not-burn ever-use were calculated by multivariable logistic regression models using covariates including socio-demographic factors. Stratified analyses according to smoking status (combustible cigarettes) were additionally performed for e-cigarette ever-use and heat-not-burn tobacco product ever-use.

**Results:**

Associations between educational attainment and e-cigarette ever-use or heat-not-burn tobacco ever-use are not straightforward, although these associations are not statistically significant except for one cell. For example, using "graduate school" education as a reference category, adjusted ORs for "high school" were 1.44 (95% confidence interval [CI]: 0.85–2.44) for e-cigarettes ever-use and 0.75 (95% CI:0.19–2.97) for heat-not-burn tobacco product ever-use. Among current smokers, compared with “graduate school” (reference), those with lower educational attainment showed 0.6 to 0.7 ORs for e-cigarette ever-use: e.g.,"4-year university"(OR = 0.54, 95% CI:0.24–1.24) and "high school" (OR = 0.69, 95% CI: 0.30–1.60). Among former smokers, lower education indicated higher ORs for both e-cigarettes and heat-not-burn tobacco ever-use.

**Conclusions:**

This study provides baseline information on educational gradients of e-cigarette and heat-not-burn tobacco products, ever-use. As heat-not-burn tobacco products are increasing their market share in Japan, continuous monitoring of these products will be necessary.

## Introduction

Combustible cigarette smoking is the most attributable and preventable risk factor for adult mortality and morbidity worldwide including Japan [1–4]. In order to promote tobacco control activities, it is important to know the social determinants of smoking. In this connection, many previous studies have reported that smoking behavior was associated with various socioeconomic positions: e.g., the prevalence of current smoking was high among persons with a low educational level, blue-collar work, and low income [5,6]. While poorly-educated people are likely to be smokers, highly-educated people may be more health conscious and therefore more likely to quit smoking [7]. As for e-cigarettes, Regan et al. [8] found that those with less than high school education were less likely to be aware of e-cigarettes than those with college level education. This may relate to the fact that businesses have promoted e-cigarettes and heat-not-burn tobacco products as smoking-cessation aids or harm-reduction products. Furthermore, the price of e-cigarettes and heat-not-burn tobacco products is usually higher than that of combustible cigarettes in Japan where the absolute cigarette price is low [9]. On the other hand, combustible cigarettes smokers, negatively correlated with education levels [10], are more likely to use e-cigarettes compared with never smokers [6, 8, 11, 12]. Educational attainment is a representative socioeconomic factor in Japan [13], which is less likely to change in adulthood [14].

Multiple tobacco products, such as combustible cigarettes, electronic cigarettes (e-cigarettes), heat-not-burn tobacco products and others are used worldwide [15, 16]. E-cigarettes are battery-powered devices that provide inhaled doses of nicotine and other additives to the user [17, 18]. They initially emerged in China in 2003 and have since become widely available globally, particularly on the internet market in Japan. Recently, new heat-not-burn tobacco products have been sold in some countries including Italy and Japan. These include brand names Ploom Tech produced by Japan Tobacco and iQOS produced by Philip Morris, which have been sold since December 2013 and November 2014, respectively, in Japan. However, to date, studies to investigate educational gradients in the use of e-cigarettes and heat-not-burn tobacco products are scarce. A few studies [11, 19] have reported the association between e-cigarette use and educational attainment but no studies have examined the educational gradients in the use of heat-not-burn tobacco products. In this study, we analyzed the association between educational attainment and use of e-cigarettes and heat-not-burn tobacco products.

## Methods

### Internet survey

The survey was conducted between 31 January and 17 February 2015 among the first 9,000 respondents (actually 9,055 including concurrent excess 55; the questionnaire took about 30 minutes to complete which meant that 55 people had begun to answer questions before the targeted number of respondents in each category was reached and continued to finish their survey); i.e., 500 people of 15–19 years for both genders and 800 people of 20–29, 30–39, 40–49, 50–59 and 60–69 years for both genders. Details are also explained elsewhere [6]. Respondents were invited to participate in the survey from a large survey panel managed by a major nationwide internet research agency, Rakuten Research [6]. The overall size of the survey panel at the time of the survey was 2,278,733 people, of whom 53.9% were male. The panel members represented all social categories (such as education, housing tenure and marital status) defined by the Census in Japan. The survey panel consisted of people initially recruited through services managed by the research agency group. At the time of registration, they were required to provide information such as gender, age, occupation and residence, and to agree that they would participate in different research surveys with web-based written consent. Minors provided their consent with approval from their parents or guardians. This study protocol was approved by the Research Ethics Committee of the Osaka Medical Center for Cancer and Cardiovascular Diseases in 2014 (No. 1412175183).

In this survey, panelists were asked about their use of e-cigarettes and heat-not-burn tobacco products with some demographic, socioeconomic and health-related factors. The survey requests were sent by the research agency to the panelists who were selected for each gender and age category using simple random sampling. Panelists who consented to participate in the survey accessed the designated website and responded to the questionnaire. Panelists had the option to not respond to any part of the questionnaire and the option to discontinue the survey at any point. The survey was closed when the target numbers of respondents for each gender and age category were met. The participation rate [17] for the survey was 8.5% (9,055/106,202). Detailed information on the study has been given in a previous publication [6].

To validate the data, questions about the total number of household members were used to detect any discrepancies in the responses. Respondents were asked the total number of household members and the number of household members in each age group separately. Thus, we could identify any discrepancies in their responses (n = 644) to assess the data quality. In addition to discrepancies, artificial responses were found in some cases (n = 274); i.e., respondents who chose all the same options in some questions that had more than ten items. Thus, we excluded respondents with discrepancies or artificial responses (n = 815) from our analyses.

### Education

Five categories of educational attainment were defined: “Junior high school (9 years of mandatory education)" comprised those who finished junior high school without graduating or were presently attending high school; "high school (12 years education)" comprised those who had graduated or who were presently attending high school without graduating or were presently taking further educational steps; "2-year college (14 years education)" comprised those who graduated or were presently attending 2-year college or technical professional school without graduating or were presently attending 4-year college; "4-year university (16 years education)" comprised those who graduated or were presently attending 4-years university without graduating or were presently attending graduate school; "graduate school (18–22 years education)" comprised those who graduated or were presently attending graduate school having previously graduated 4-years university. These definitions would include some misclassifications, but might be reasonable because of low drop-out rate in Japan.

### Combustible cigarette (smoking status)

Panelists were also asked, "Please choose your current status for paper-wrapped and roll-your-own cigarette separately." The response options were "never user", "former non-regular user", "former regular user" and "current user". Respondents who currently smoked combustible tobacco (paper-wrapped and/or roll-your-own cigarette) at the time of the survey were considered current smokers. Those who reported former use and did not currently smoke either type of cigarette were considered former smokers. Those who had never smoked were considered never smokers.

### E-cigarettes and heat-not-burn tobacco products in Japan

Several types of e-cigarettes and heat-not-burn tobacco products were available in 2015 in Japan [6]. Although the sale of e-cigarettes containing nicotine has been banned since 2010 by the Pharmaceutical Affairs Act, e-cigarettes that do not contain nicotine are not legally regulated. Therefore, the use of e-cigarettes containing nicotine in Japan is limited to privately imported products, whereas e-cigarettes that do not contain nicotine are widely sold in Japanese marketplaces, even to minors. In December 2013, Japan Tobacco, a tobacco company in Japan, started to sell a new heat-not-burn tobacco product, Ploom, which vaporizes tobacco leaves rather than a liquid. Philip Morris also developed a new heat-not-burn tobacco product, iQOS, which heats tobacco leaves, and introduced it to the market in November 2014. Ploom and iQOS are legally regarded as tobacco products and are regulated by the Tobacco Industries Act. Panelists were asked about their use of each of the following products: nicotine e-cigarettes, non-nicotine e-cigarettes, e-cigarettes with unknown nicotine content, Ploom and iQOS, using the question, "Please choose your current status for each product." and the response options were "never user", "former non-regular user", "former regular user" and "current user". The latter three responses were combined and defined as "ever user" of each product. Respondents who reported ever-use (at least once) of at least one type of product (of the above-mentioned former three e-cigarettes) were considered ever-users of e-cigarettes. Ever-use of heat-not-burn tobacco products was defined as ever-use of Ploom and/or iQOS to obtain an estimate for heat-not-burn tobacco products.

### Other variables

Data for age group (18–24 years, 25–29, 30–39, 40–49, 50–59, 60–69), gender (men or women), marital status (married or non-married) and self-rated health (good or poor) were used as covariates. Married people were those married at the time of the survey. Non married was defined as people who had never been married, were divorced or whose spouse had died. Self-rated health was dichotomized: good (excellent, very good, or good) or poor (fair or poor).

### Statistical analysis

In this study, adults aged 18–69 years were analyzed. People whose educational status was missing were excluded from the analysis. Differences in characteristics according to educational attainment were examined using the chi-squared test.

Logistic regression models were used to calculate the odds ratios (ORs) and 95% confidence intervals (CIs) for current smoking, e-cigarette ever-use, or heat-not-burn tobacco product ever-use. Multivariable analyses were used to document the adjusted relationship between educational attainment and the above-mentioned three outcomes. In addition to the total sample analysis, stratified analyses according to smoking status (current, former and ever smokers) were performed for e-cigarette ever-use and heat-not-burn tobacco product ever-use. All analyses were performed using SAS version 9.3 (SAS Institute Inc., Cary, NC, USA) and Matlab version R2016a (The MathWorks Inc., Natick, Massachusetts, USA).

## Results

After excluding respondents with a discrepancy, those under 25 years old and those with missing educational attainment, 7338 subjects remained. Table 1 shows the basic characteristics of the study subjects according to educational attainment. Distribution of all the factors significantly differed according to educational attainment: for example, women comprised 49.1% of junior high school graduates, 72.6% of 2-year college graduates, and 26.6% of graduate school graduates.

**Table 1:** Basic characteristics according to educational attainment.

	Total	Junior high school	High school	2-year college	4-year university	Graduate school	
Variables	No (%)	No (%)	No (%)	No (%)	No (%)	No (%)	P for difference*
**Total**	7338 (100.0)	161 (2.2)	2038 (27.8)	1641 (22.4)	3095 (42.2)	403 (5.5)	
**Age group, years**							
18–24	397 (5.4)	17 (10.6)	90 (4.4)	51 (3.1)	220 (7.1)	19 (4.7)	<0.001
25–29	1058 (14.4)	29 (18.0)	241 (11.8)	197 (12.0)	495 (16.0)	96 (23.8)	
30–39	1461 (19.9)	37 (23.0)	334 (16.4)	357 (21.8)	617 (19.9)	116 (28.8)	
40–49	1482 (20.2)	31 (19.3)	430 (21.1)	395 (24.1)	554 (17.9)	72 (17.9)	
50–59	1459 (19.9)	18 (11.2)	425 (20.9)	352 (21.5)	600 (19.4)	64 (15.9)	
60–69	1481 (20.2)	29 (18.0)	518 (25.4)	289 (17.6)	609 (19.7)	36 (8.9)	
**Gender**							<0.001
Men	3632 (49.5)	82 (50.9)	931 (45.7)	449 (27.4)	1874 (60.5)	296 (73.4)	
Women	3706 (50.5)	79 (49.1)	1107 (54.3)	1192 (72.6)	1221 (39.5)	107 (26.6)	
**Marital status**							<0.001
Married	4537 (61.8)	95 (59.0)	1256 (61.6)	1062 (64.7)	1919 (62.0)	205 (50.9)	
Non-Married	2801 (38.2)	66 (41.0)	782 (38.4)	579 (35.3)	1176 (38.0)	198 (49.1)	
**Self-rated health**							<0.001
Good	6487 (88.4)	126 (78.3)	1764 (86.6)	1464 (89.2)	2766 (89.4)	367 (91.1)	
Poor	851 (11.6)	35 (21.7)	274 (13.4)	177 (10.8)	329 (10.6)	36 (8.9)	

*P value was calculated by chi-square test.

Table 2 shows the percentage (%) of current smokers, e-cigarette ever users, and heat-not-burn tobacco ever users according to characteristics including educational attainment. A significant inverse association between educational attainment and current smoking was observed: using "graduate school" education as a reference category, OR for ‘junior high school’ was 5.65 (95% CI: 3.36–9.50), OR for ‘high school’ was 3.34 (2.27–4.91), and OR for ‘4-year university’ was 2.20 (1.51–3.22). On the other hand, the association between educational attainment and e-cigarette ever-use or heat-not-burn tobacco product ever-use was not significant: for example, ORs for "high school" were 1.44 (0.85–2.44) for e-cigarettes ever-use and 0.75 (0.19–2.97) for heat-not-burn tobacco product ever-use. Among e-cigarette ever users (n = 394), more than half (51.3%) subjects were current smokers (n = 202). Also, among heat-not-burn tobacco products ever-users (n = 40), approximately half (47.5%) of all subjects were current smokers (n = 19).

**Table 2:** Odds ratios (95% CI) for current smoking, e-cigarette ever-use and heat-not-burn tobacco product ever-use.

Characteristics	Total	Current smoking (combustible cigarette)		E-cigarette ever use		Heat-not-burn tobacco product ever use	
	No (%)	No (%)	OR* (95% CI)	No (%)	OR† (95% CI)	No (%)	OR† (95% CI)
**Total**	7338	1080 (14.7)		394 (5.4)		40 (0.5)	
**Educational attainments**							
Junior high school	161 (2)	42 (26.1)	5.65 (3.36–9.50)	18 (11.2)	1.74 (0.84–3.62)	2 (1.2)	1.46 (0.22–9.80)
High school	2038 (28)	364 (17.9)	3.34 (2.27–4.91)	141 (6.9)	1.44 (0.85–2.44)	8 (0.4)	0.75 (0.19–2.97)
2-year college	1641 (22)	205 (12.5)	2.62 (1.76–3.91)	81 (4.9)	1.24 (0.71–2.16)	7 (0.4)	0.99 (0.24–4.07)
4-year university	3095 (42)	437 (14.1)	2.20 (1.51–3.22)	136 (4.4)	0.89 (0.52–1.50)	20 (0.6)	1.12 (0.32–3.96)
Graduate school	403 (6)	32 (7.9)	1 (reference)	18 (4.5)	1 (reference)	3 (0.7)	1 (reference)
**Age group, years**							
18–24	397 (5.4)	31 (7.8)	0.58 (0.38–0.88)	28 (7.1)	1.41 (0.86–2.30)	5 (0.0)	0.98 (0.35–2.78)
25–29	1058 (14.4)	116 (11.0)	1 (reference)	71 (6.7)	1 (reference)	18 (1.7)	1 (reference)
30–39	1461 (19.9)	213 (14.6)	1.41 (1.10–1.81)	91 (6.2)	0.71 (0.50–1.00)	10 (0.7)	0.29 (0.13–0.64)
40–49	1482 (20.2)	269 (18.2)	1.81 (1.42–2.31)	80 (5.4)	0.48 (0.34–0.69)	1 (0.1)	0.02 (0.00–0.17)
50–59	1459 (19.9)	271 (18.6)	1.97 (1.53–2.52)	62 (4.2)	0.36 (0.25–0.53)	4 (0.3)	0.08 (0.02–0.25)
60–69	1481 (20.2)	180 (12.2)	1.15 (0.88–1.50)	62 (4.2)	0.40 (0.27–0.59)	2 (0.1)	0.04 (0.01–0.18)
**Gender**							
Men	3632 (49.5)	756 (20.8)	1 (reference)	256 (7.0)	1 (reference)	31 (0.9)	1 (reference)
Women	3706 (50.5)	324 (8.7)	0.33 (0.29–0.39)	138 (3.7)	0.79 (0.63–1.01)	9 (0.2)	0.43 (0.20–0.96)
**Marital status**							
Married	4537 (61.8)	643 (14.2)	0.79 (0.68–0.92)	234 (5.2)	1.11 (0.88–1.41)	20 (0.4)	1.32 (0.65–2.70)
Non-Married	2801 (38.2)	437 (15.6)	1 (reference)	160 (5.7)	1 (reference)	20 (0.7)	1 (reference)
**Self-rated health**							
Good	6487 (88.4)	938 (14.5)	1 (reference)	347 (5.3)	1 (reference)	36 (0.6)	1 (reference)
Poor	851 (11.6)	142 (16.7)	1.05 (0.86–1.28)	47 (5.5)	1.00 (0.72–1.40)	4 (0.5)	0.96 (0.33–2.77)
**Combustible cigarette**							
Never user	4546 (62.0)	NA	NA	72 (1.6)	1 (reference)	4 (0.1)	1 (reference)
Former user	1712 (23.3)	NA	NA	120 (7.0)	5.28 (3.87–7.19)	17 (1.0)	16.91 (5.56–51.50)
Current user	1080 (14.7)	NA	NA	202 (18.7)	15.24 (11.36–20.46)	19 (1.8)	25.73 (8.50–77.88)

*Adjusted for age, gender, marital status and self-rated health.

^†^Adjusted for age, gender, marital status, self-rated health and smoking status (combustible cigarette).

OR: odds ratio, E-cigarette: electronic cigarette, No: number, NA: not applicable.

ORs of educational categories for e-cigarette ever-use or heat-not-burn tobacco ever-use among current, former or ever smokers are shown in Table 3 (stratified analyses). ORs of education categories for e-cigarette ever-use and heat-not-burn tobacco ever-use were not straightforward. No significant results were not observed for heat-not-burn tobacco products, probably because sample size and prevalence of the products were considerably low. Compared with graduate school graduates, other lower educational levels indicated non-significant but less than one values of ORs for both e-cigarette ever-use and heat-not-burn tobacco product ever-use among current smokers. Inversely, lower educational levels, especially "junior high school" graduates, indicated higher values of ORs for both ever-use among former smokers, although statistical significance was not observed partly due to small sample size.

**Table 3:** Odds ratios (95% CI) of educational attainment for ever-use of e-cigarette and heat-not-burn tobacco products.

	Total	E-cigarette ever-use		Heat-not-burn tobacco product ever-use	
	No (%)	No (%)	OR* (95% CI)	No (%)	OR* (95% CI)
**Current smokers**					
Junior high school	42 (3.9)	8 (19.0)	0.62 (0.20–1.88)	1 (2.4)	0.50 (0.04–6.54)
High school	364 (33.7)	69 (19.0)	0.69 (0.30–1.60)	1 (0.3)	0.07 (0.01–0.85)
2-year college	205 (19.0)	41 (20.0)	0.75 (0.31–1.78)	5 (2.4)	0.63 (0.11–3.65)
4-year university	437 (40.5)	70 (16.0)	0.54 (0.24–1.24)	10 (2.3)	0.52 (0.10–2.59)
Graduate school	32 (3.0)	9 (28.1)	1 (reference)	2 (6.3)	1 (reference)
**Ever smokers**					
Junior high school	74 (2.7)	14 (18.9)	1.85 (0.82–4.19)	2 (2.7)	1.56 (0.23–10.45)
High school	844 (30.2)	112 (13.3)	1.50 (0.83–2.69)	7 (0.8)	0.64 (0.16–2.62)
2-year college	535 (19.2)	65 (12.1)	1.34 (0.72–2.47)	7 (1.3)	1.00 (0.24–4.12)
4-year university	1203 (43.1)	116 (9.6)	1.02 (0.57–1.82)	17 (1.4)	0.99 (0.28–3.55)
Graduate school	136 (4.9)	15 (11.0)	1 (reference)	3 (2.2)	1 (reference)
**Former smokers**					
Junior high school	32 (1.9)	6 (18.8)	3.14 (0.87–11.34)	1 (3.1)	3.88 (0.20–75.84)
High school	480 (28.0)	43 (9.0)	1.83 (0.74–4.53)	6 (1.3)	2.26 (0.25–20.25)
2-year college	330 (19.3)	22 (6.7)	1.29 (0.49–3.40)	2 (0.6)	1.12 (0.09–13.49)
4-year university	766 (44.7)	43 (5.6)	1.13 (0.46–2.77)	7 (0.9)	1.60 (0.19–13.81)
Graduate school	104 (6.1)	6 (5.8)	1 (reference)	1 (1.0)	1 (reference)

*Adjusted for Age, Gender, Marital status, and Self-rated health.

OR: odds ratio, E-cigarette: electronic cigarette, No: number, NA: not applicable.

Stratified analyses among current, former or ever smokers.

## Discussion

In this study, we confirmed an inverse association between educational attainment and combustible cigarette smoking. This is not surprising as this association has been shown in many previous studies [5, 10, 13]. However, in contrast with this, educational levels were not clearly associated with e-cigarette ever-use and heat-not-burn tobacco ever-use: for example, compared with "graduate school" graduates, "high school" graduates were likely to use e-cigarettes, but not heat-not-burn tobacco products, although the ORs were not statistically significant.

It is important to consider a power of analysis, especially when dealing with small subjects with low prevalence. In the current study, there are 403 subjects in graduate school (reference category), 3095 subjects in 4-year university and 161 subjects in junior high school (Table 2). Analyzing these subjects, educational gradients for combustible cigarette smoking were clearly observed. The ORs for current smoking were significant, ranging from 2.20 to 5.65. Under this condition, power analysis indicated a power of more than 99% for subjects in junior high school (n = 161, OR = 5.65). Even when OR = 3, power became slightly lower 98%. Similarly, power was estimated 99% for subjects in 4-year university (n = 3095, OR = 2.20). Next, for heat-not-burn tobacco ever use, power analysis was performed, assuming that educational gradients are equivalent to the case of combustible cigarette smoking. Power analysis indicated a power of 82% for subjects in junior high school (n = 161, OR = 5.65) and 30% for subjects in 4-year university (n = 3095, OR = 2.20). If the same educational gradients as combustible cigarette was also observed for heat-not-burn tobacco ever use, we can assume a certain level of power in the analysis. However, educational gradients in heat-not-burn tobacco was not large, and thus resulted in no significance. Because heat-not-burn tobacco products recently become more popular in Japan [20], we need to continue monitoring for educational gradients in heat-not-burn tobacco use in Japan.

In the stratified analyses, compared with graduate school graduates (reference), those with lower education levels had negative tendency not to use either e-cigarettes or heat-not-burn tobacco products among current smokers, while junior high school graduates had positive tendency to use both among former smokers. This is the first study, to our knowledge, to estimate educational gradients of use of heat-not-burn tobacco products.

Heat-not-burn tobacco products are sold in only a few countries including Japan. While this product shares common features with e-cigarettes, it is defined as a tobacco product containing tobacco leaves as a raw material in accordance with the tobacco business law in Japan. E-cigarettes with nicotine are prohibited by the Pharmaceutical Affairs Law in Japan. Therefore, it can be seen that Japan is being used as an experimental site for the marketing of heat-not-burn tobacco products by the tobacco industry in an environment where the use of e-cigarettes containing nicotine has not spread widely [6]. Recently, the British American Tobacco started to sell a new heat-not-burn tobacco product named "glo" in Japan. Because heat-not-burn tobacco products are taking an increasingly large share of the market only in Japan (Japanese share of iQOS was reported to account for about 98% worldwide by a Japanese newspaper in December 2016, citing a comment of a person who was working in the Philip Morris Japan), continuous monitoring of these products will be necessary.

### Limitations

There are several limitations to this study. First, since it is based on an internet survey, the results of this study may be different from those from a population-based study: i.e., the generalizability of the study to the general population should be considered carefully. However, because many previous studies for e-cigarette use have been conducted using internet surveys [21] and electronic cigarettes are sold mostly via the internet, especially nicotine-containing e-cigarettes in Japan, an internet survey may be appropriate to investigate e-cigarette use. Second, this survey might underestimate “ever use” or “former use”, because respondents might misclassify themselves. For instance, a respondent who has only smoked a few cigarettes in their life might be categorized as “never user” rather than “former user.” Furthermore, some respondents might use a friend’s product without buying their own e-cigarette or heat-not-burn tobacco products and not consider themselves “former users”. Furthermore, we expected that people might not distinguish heat-not-burn tobacco products with e-cigarettes. This might overestimate prevalence of e-cigarettes. Finally, the number of heat-not-burn tobacco products ever-use observed in the study was considerably lower than that of combustible tobacco products. However, because of the rapid spread of heat-not-burn tobacco products reported in Japan, this low prevalence of heat-not-burn tobacco products may contribute to future study as baseline data in 2015.

## Supporting information

S1 Text The names of the variables and their details. Bold means categories’ name.
(DOCX)

S1 Table Raw data according to the characteristics. Each characteristic is defined in S1 Text.
(CSV)
