# Supplementary material for: Educational gradients in the use of electronic cigarettes and heat-not-burn tobacco products in Japan
Source: PLoS One. 2018 Jan 12;13(1):e0191008. doi: 10.1371/journal.pone.0191008 (PMC5766136; doi:10.1371/journal.pone.0191008)
Supplement: S1 Text — Bold means categories’ name. (DOCX) [file pone.0191008.s001.docx]

Data Availability Statement: The data set analyzed in the article is available as a Supporting Information file.

S1 Text: explanation of data (variable name and the code)

S1 Table is the anonymized data set analyzed in the article “Educational gradients in the use of electronic cigarettes and heat-not-burn tobacco products in Japan.”

Followings are the name of the variables and their details.

**Bold** means categories’ name.

・**Age**: means Age group, years

1 (18-24), 2 (25-29), 3 (30-39), 4 (40-49), 5 (50-59), 6 (60-69)

・**Gender**: means Gender

1 (Men), 2 (Women)

・**Education**: means Educational attainments

1 (Junior high school), 2 (High school), 3 (2-year college), 4 (4-year university), 5 (Graduate school)

・**Smoking**: means Smoking status

0 (Never user), 1 (Former user), 2 (Current user)

*Ever user means Former user or Current user.

・**Ecig**: means E-cigarettes using status

0 (Never user), 1 (Ever user)

・**Heatnotburn**: means heat-not-burn tobacco using status

0 (Never user), 1 (Ever user)

・**Marry**: means marital status

0 (non-married), 1 (married)

・**Health**: means Self-rated health

0 (poor), 1 (good)
